# An Improved Method for Rapid Detection of *Mycobacterium abscessus* Complex Based on Species-Specific Lipid Fingerprint by Routine MALDI-TOF

**DOI:** 10.3389/fchem.2021.715890

**Published:** 2021-07-27

**Authors:** Min Jia Khor, Agnieszka Broda, Markus Kostrzewa, Francis Drobniewski, Gerald Larrouy-Maumus

**Affiliations:** ^1^ MRC Centre for Molecular Bacteriology and Infection, Department of Life Sciences, Faculty of Natural Sciences, Imperial College London, London, United Kingdom; ^2^ Department of Infectious Diseases, Faculty of Medicine, Imperial College London, London, United Kingdom; ^3^ Bruker Daltonics GmbH & Co. KG, Bremen, Germany

**Keywords:** MALDI, lipids, diagnostics, mycobacteria, solvent

## Abstract

Rapid diagnostics of bacterial infection is the key to successful recovery and eradication of the disease. Currently, identification of bacteria is based on the detection of highly abundant proteins, mainly ribosomal proteins, by routine MALDI-TOF mass spectrometry. However, relying solely on proteins is limited in subspecies typing for some pathogens. This is the case for, for example, the mycobacteria belonging to the *Mycobacterium abscessus* (MABS) complex, which is classified into three subspecies, namely, *M. abscessus* subsp. *abscessus*, *M. abscessus* subsp. *bolletii*, and *M. abscessus* subsp. *massiliense*. Being able to detect bacteria accurately and rapidly at the subspecies level could not only reliably identify the pathogen causing the disease but also enable better antibiotic stewardship. For instance, *M. abscessus* subsp. *abscessus* and *M. abscessus* subsp. *bolletii* possess a functional erm41 (erythromycin ribosomal methylation gene 41) gene, whilst *M. abscessus* subsp. *massiliense* does not, resulting in differences in macrolide antibiotic (e.g., clarithromycin and azithromycin) susceptibilities. This presents a challenge for physicians when designing an appropriate treatment regimen. To address this challenge, in addition to proteins, species-specific lipids have now been considered as a game changer in clinical microbiology diagnostics. However, their extraction can be time-consuming, and analysis requires the use of apolar toxic organic solvents (e.g., chloroform). Here, we present a new method to accurately detect species and subspecies, allowing the discrimination of the mycobacteria within the MABS complex and relying on the use of ethanol. We found that a combination of the matrix named super-DHB with 25% ethanol with a bacterial suspension at McFarland 20 gave robust and reproducible data, allowing the discrimination of the bacteria within the MABS complex strains tested in this study (*n* = 9). Further investigations have to be conducted to validate the method on a larger panel of strains for its use in diagnostic laboratories.

## Introduction

Matrix-assisted laser desorption ionization/time-of-flight (MALDI-TOF) mass spectrometry (MS), a cost-effective, rapid, and accurate method for microbial identification based on protein signatures, has revolutionized microbiological diagnostics (Croxatto et al., 2012; Clark et al., 2013; Singhal et al., 2015). However, protein profiling by MALDI-TOF MS possesses some limitations when it comes to an efficient typing for some closely related species and subspecies (Kostrzewa et al., 2019; Welker et al., 2019).

That is the case for the bacterium named *Mycobacterium abscessus*, which is part of a complex of very similar organisms. The *M. abscessus* complex (MABS complex) is classified into three subspecies, namely, *M. abscessus* subsp. *abscessus*, *M. abscessus* subsp. *bolletii*, and *M. abscessus* subsp. *massiliense*. *M. abscessus* is found to be present within 80% of the respiratory isolates of rapidly growing mycobacteria and demonstrates resistance to many antibiotic classes like aminoglycosides, β-lactams, and macrolides (Johansen et al., 2020). For example, *M. abscessus* subsp. *abscessus* and *M. abscessus* subsp. *bolletii* possess a functional erm41 (erythromycin ribosomal methylation gene 41) gene, whilst *M. abscessus* subsp. *massiliense* does not (Kim et al., 2010; Ryu et al., 2016; Ryan and Byrd, 2018). This results in different macrolide antibiotic (i.e., clarithromycin and azithromycin) susceptibilities. Therefore, identification of the different subspecies is important in the clinical environment. As mentioned earlier, clarithromycin is more effective against *M. abscessus* subsp. *massiliense* lung infections, while resistance is commonly observed in *M. abscessus* subsp. *abscessus* and *M. abscessus* subsp. *bolletii* isolates (Adekambi et al., 2006; Esther et al., 2010; Kim et al., 2010; Koh et al., 2011; Harada et al., 2012). This presents a challenge for physicians when designing an appropriate treatment regimen (Koh et al., 2011; Brown-Elliott et al., 2012; Harada et al., 2012; Kothavade et al., 2013).

Current available tools, such as PCR, sequencing of multiple genes, and whole genome sequencing, are time-consuming, relatively expensive, and require experts for the sample preparation and data analysis, which is not ideal for routine clinical use despite offering excellent subspecies identification (Nash et al., 2009; Kim et al., 2010; Koh et al., 2011). That is why having a diagnostic tool that allows early MABS complex identification would be ideal for subspecies identification and therefore guiding drug prescription (Ryu et al., 2016). In order to introduce this tool across clinical laboratories worldwide, it needs to be simple, rapid, inexpensive, and highly specific. Therefore, the continuous development of laboratory techniques for subspecies-level discrimination between bacterial species alongside the identification of their antibiotic-resistant counterparts is of utmost importance to best assist physicians in the administration of an appropriate drug regimen.

MALDI-TOF MS offers an alternative to the methods cited earlier. Indeed, several studies report on the use of MALDI-TOF MS to detect bacteria within the MABS complex (Tseng et al., 2013; Fangous et al., 2014; Panagea et al., 2015; Kehrmann et al., 2016; Luo et al., 2016). Those approaches rely on a multistep sample preparation consisting of the inactivation of the bacteria, followed by mechanical lysis in order to have access to intracellular proteins that serve as markers. However, the multistep strategy combined with extensive data analysis used to detect the MABS complex can be improved in order to ease routine microbiology laboratory practice.

In terms of addressing this unmet need, species-specific lipid fingerprints could represent an attractive approach when it comes to microbial identification by MALDI-ToF MS. Lipids are highly abundant in bacteria and structurally diverse. Several studies have reported successfully on the use of microbial lipidomics by MALDI-ToF MS and their uses in academic laboratories and diagnostic laboratories for accurate microbial identification, making lipid fingerprinting a complementary approach to protein-based fingerprinting in order to tackle the challenge of microbial differentiation by MALDI-ToF MS (Shu. et al., 2012; Voorhees et al., 2013; Cox et al., 2015; Larrouy-Maumus and Puzo, 2015; Leung et al., 2017; Gonzalo et al., 2020; Lellman and Cramer, 2020).

Nevertheless, those methods, based on lipid fingerprints (cited earlier), rely on the use of highly toxic organic solvents (e.g., CHCl_3_, CH_3_OH, or a mixture of CHCl_3_ and CH_3_OH), hampering an easy implementation of the method in routine clinical laboratories due to safety concerns. Also, those solvents have a relative polarity ranging from 0.259 to 0.762, which could have an impact on the selectivity of the species-specific lipids to be extracted and detected by MALDI-TOF MS, influencing the outcome of bacterial identification. To address this challenge, we investigated the use of ethanol (CH_3_CH_2_OH, relative polarity 0.654) as a commonly used organic solvent widely used in routine clinical microbiology laboratories worldwide as an alternative to chloroform and methanol to extract species-specific lipids for the rapid identification of bacteria belonging to the MABS complex.

## Materials and Methods

### Bacterial Strains and Growth Conditions

*M. abscessus* subsp. *abscessus* ATCC 19977, *M. abscessus* subsp. *abscessus* M02004436, *M. abscessus* subsp. *abscessus* M07001466, *M. abscessus* subsp. *bolletii* M09013950, *M. abscessus* subsp. *bolletii* M04009973, *M. abscessus* subsp. *bolletii* H123680049, *M. abscessus* subsp. *massiliense* M10010169, *M. abscessus* subsp. *massiliense* M07012434, and *M. abscessus* subsp. *massiliense* H120280022 obtained from the National Mycobacterium Reference-NMRS-South (United Kingdom) were cultured in Middlebrook 7H9 broth liquid medium supplemented with 0.5 g/L Fraction V (bovine serum albumin), 0.05% tyloxapol, 0.2% dextrose, 0.2% glycerol, and 10 mM NaCl. The inoculated cultures were then incubated at 37^°^C for 2 days. Bacterial pellets were heat-killed at 95°C for 30 min before leaving the BSL3 containment area.

### Sample Preparation

**FIGURE 1:**
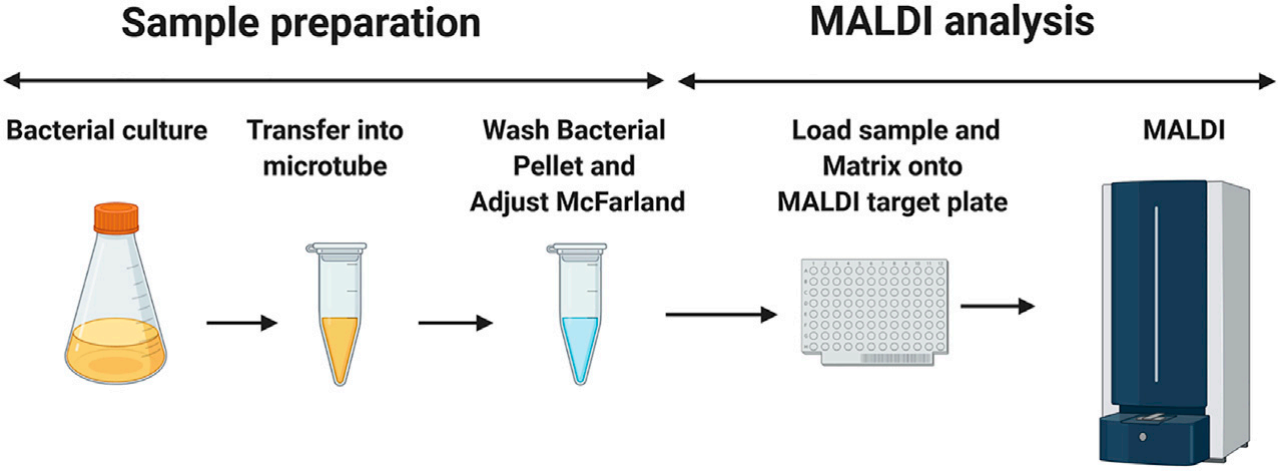
Schematic diagram of the sample preparation before the MALDI mass spectrometry measurement. 1.5 ml of mycobacterial culture is aliquoted in a microtube. The mycobacterial suspension is then washed twice with double-distilled water and adjusted to the desired McFarland suspension. 0.5 μL of this preparation is loaded into the MALDI target plate, followed by the addition of 1.2 μl of the matrix (super-DHB solubilized at 10 mg/ml in ethanol) and mixing on the MALDI MBT 96 Biotarget plate. Once dried, the mass spectra are acquired in the linear positive ion mode. The image has been created using BioRender.

100 µl of bacterial suspension (from liquid- or agar-based media) was placed into 1.5 ml microtubes that contain 200 µl of double-distilled water. The bacteria were then washed twice with 200 μl of double-distilled water and suspended at various McFarland standards ranging from 5 to 40 using a McFarland tube densitometer (Grant-Bio®). Then, 0.5 µl of the resuspended pellet was pipetted onto the MALDI target plate and mixed with 1.2 µl of the MALDI matrix (Figure 1). The matrix used consisted of a 9:1 mixture of 2,5-dihydroxybenzoic acid and 2-hydroxy-5-methoxybenzoic acid (super-DHB, Sigma-Aldrich) at a concentration of 10 mg/ml in ethanol at various percentages ranging from 0 to 100%. Additionally, for external calibration, 0.5 µl of calibration peptide mixture was loaded along with 0.5 µl of the given calibration matrix (peptide calibration standard II, Bruker Daltonics, Germany). The samples were loaded onto a disposable MBT 96 Biotarget plate (Bruker Part-No. 1840375).

### Mass Spectrometry Analysis

MS analyses were performed on an MALDI Biotyper Sirius system (Bruker Daltonics, Germany). The mass spectra were scanned in the range of m/z 1,100–1,600. The mass profiles were acquired using FlexControl 3.4 software (Bruker Daltonics, Germany). The spectra were recorded in the linear positive ion mode (laser intensity 95%, ion source 1 = 10.00 kV, ion source 2 = 8.98  kV, lens = 3.00 kV, detector voltage = 2652 V, pulsed ion extraction = 150 ns). Each spectrum corresponded to an ion accumulation of 5,000 laser shots randomly distributed on the spot. The spectra obtained were processed with default parameters using FlexAnalysis v.3.4 software (Bruker Daltonics, Germany).

## Results and Discussion

In this study, we optimized the number of bacteria loaded onto the MALDI target plate and the solvent to solubilize the matrix prior to MS analyses in the positive ion mode to address the major challenge in subspecies typing by MALDI-TOF MS inside the MABS complex.

**FIGURE 2:**
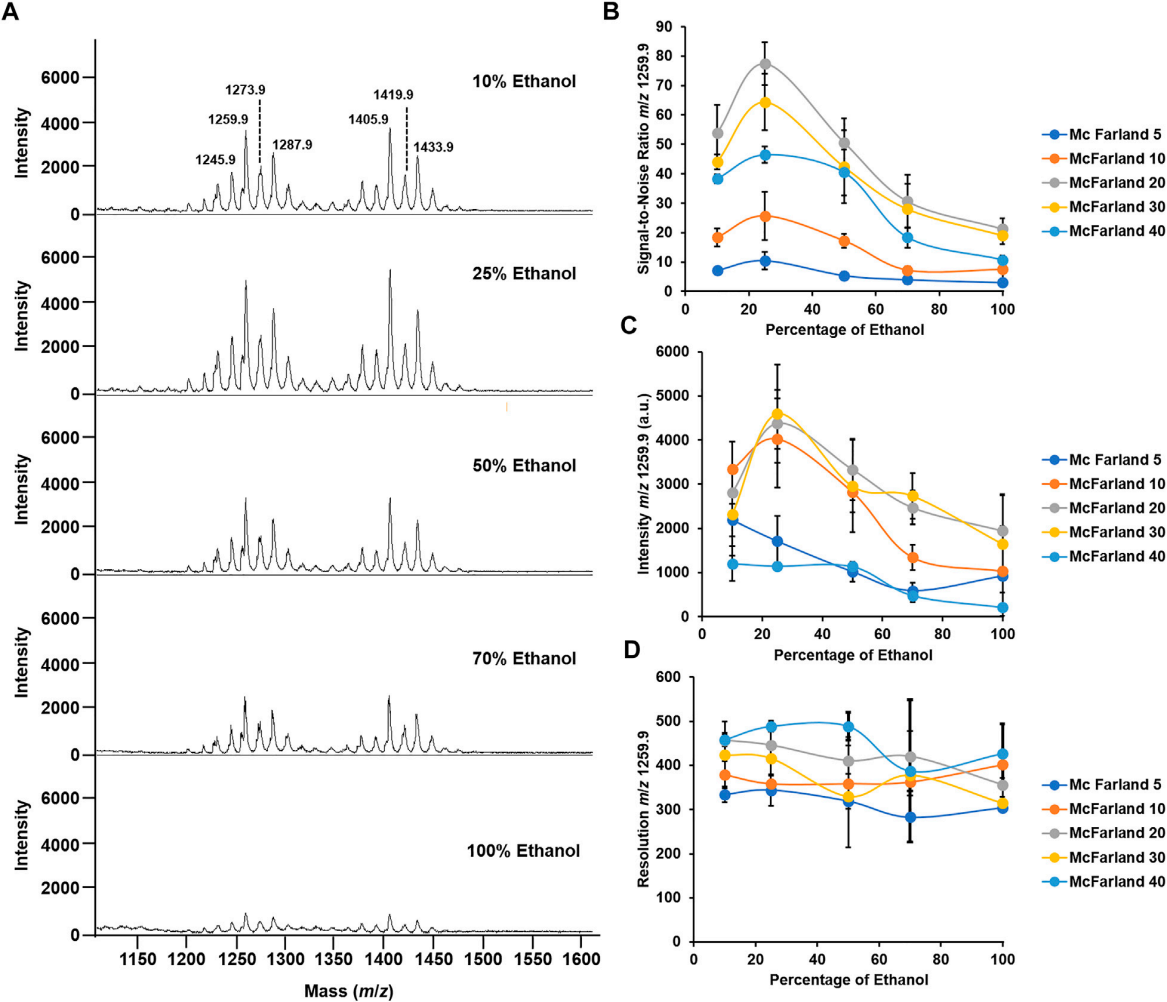
Optimization of McFarland and percentage of ethanol for the optimal mass spectrum signal. **(A)** Mass spectra acquired in the positive ion mode of *M. abscessus* subsp. *abscessus* ATCC 19977 set at McFarland 20 and for a percentage of ethanol to solubilize the matrix ranging from 10 to 100%. (**B**) Graph depicting the changes in the S/N ratio in the function of the percentage of ethanol across 5 McFarland dilutions. (**C**) Graph depicting the changes in the intensity of the peak at *m*/*z* 1259.9 in the function of the percentage of ethanol across 5 McFarland dilutions. (**D**) Graph depicting the changes in the resolution of the peak at *m*/z 1259.9 in the function of the percentage of ethanol across 5 McFarland dilutions. Experiments have been performed in biological and technical triplicates. Error bars indicate the standard deviation.

By using the reference strain *M. abscessus* subsp. *abscessus* ATCC 19977, we found that a suspension of bacteria at McFarland 20 combined with the matrix super-DHB solubilized in 25% ethanol at a final concentration of 10 mg/ml was most appropriate for our experiments. We chose to use super-DHB as a matrix due to its versatility for the analysis of lipids and glycolipids (Schiller et al., 2007). This was decided following observation of the raw mass spectra obtained across 5 McFarland dilutions (5, 10, 20, 30, and 50) and 5 percentages of ethanol used to solubilize the matrix (10, 25, 50, 70, and 100%). Regardless of the McFarland dilution and percentage of ethanol used in the study, the positive ion mass spectra of *M. abscessus* subsp. *abscessus* ATCC 19977 showed two sets of peaks starting at *m*/*z* 1201.8 up to *m*/*z* 1299.9 and starting at *m*/*z* 1375.9 up to *m*/*z* 1445.9, distant of 14 mass units with the most intense peaks at *m*/*z* 1259.9 and *m*/*z* 1405.9 assigned to potassium cationized–diglycosylated glycopeptidolipids ([M + K]^+^ *m*/*z* 1259.9) and triglycosylated glycopeptidolipids ([M + K]^+^ *m*/*z* 1405.9) (Figure 2A), which are known to make up more than 70% of the surface-exposed mycobacterial lipids. Glycopeptidolipids have been demonstrated to play a key role in mycobacterial physiology and pathogenicity (Byrd and Lyons, 1999; Howard et al., 2006; Catherinot et al., 2007; Ripoll et al., 2007; Whang et al., 2017; Gutierrez et al., 2018; Tran et al., 2019; Kurz and Rivas-Santiago, 2020; Jackson et al., 2021). Such lipid profiles are similar in terms of lipid species detected to the ones that have been obtained in the literature using conventional methods (Ripoll et al., 2007; Gonzalo et al., 2020); this indicates that as a solvent (to solubilize the matrix), ethanol produces efficient extraction and co-crystallization, and thereby desorption and ionization of surface lipids on the MALDI target plate. A possible mechanism is that ethanol could act as an eraser enabling the selective on-target extraction of the surface-exposed di- and triglycosylated glycopeptidolipids and their co-crystallization with the matrix, allowing their desorption and ionization.

In addition, here, we aimed to have the highest quality signal in order to enhance reproducible and robust data. To decide which bacterial suspension and percentage of ethanol were appropriate for performing the analysis, we recorded the signal-to-noise (S/N), resolution, and intensity of the dominant peak at *m*/*z* 1259.9. First, regarding the S/N, as seen in Figure 2B, at McFarland 5, the S/N is ∼10 across all percentages of ethanol tested. Interestingly, when using a bacterial suspension of McFarland 10, the S/N increases when passing from 10 to 25% ethanol, going from S/N 18 to 25, and then decreases and plateaus at S/N 7.5 for a percentage of ethanol of 100%. A similar pattern is observed when using higher McFarland values. Indeed, for a bacterial suspension set at McFarland 20, at 10% ethanol, the S/N is ∼50, which is almost 3 times higher than it is when using McFarland 10, and it even increases up to S/N ∼80 for a value of 25% ethanol before decreasing gradually to S/N ∼20 for 100% ethanol. However, when using higher bacterial suspension, for example, McFarland 30 or 40, the S/N tends to decrease across all percentages of ethanol tested compared to the S/N observed for McFarland 20, going for S/N to at 25% ethanol to S/N ∼60 and S/N ∼40 for the same percentage of ethanol. Such observations could be explained by the ratio of the matrix to the sample required for the optimal co-crystallization of the molecules of interest with the matrix on the MALDI target plate and allowing their optimal desorption and ionization (Smolira and Wessely-Szponder, 2015; Wiangnon and Cramer, 2015; Wang et al., 2016; Boesl, 2017). In other words, the fact that higher McFarland or higher percentage of ethanol did not generate good quality spectra could be explained by the poor co-crystallization with the matrix. That could lead to a nonhomogeneous spot on the MALDI target plate, precluding any transfer of the energy provided by the laser to the matrix in allowing the desorption of the molecules of interest (Dreisewerd, 2003; Wang et al., 2016).

A similar conclusion can be drawn for the intensity of the signal. As seen in Figure 2C, for both McFarland 5 and McFarland 40, the signal intensity remains low across all the percentages of ethanol tested, with an intensity of ∼1,000 a.u. However, for intermediate McFarland values, when going from 10% ethanol to 25% ethanol, the intensity of the signal increases from ∼2,000 a.u. to 5,000 a.u. and then decreases gradually when reaching 100% ethanol. That agrees with the S/N data where McFarland 20 and 25% ethanol are the parameters required for the optimal quality signal.

Regarding the resolution, neither McFarland nor the percentage of ethanol seems to influence that parameter which has its range from 300 to 500 with no significative differences (Figure 2D).

Taken together, a bacterial suspension set at McFarland 20 and the use of the super-DHB solubilized at 10 mg/ml in 25% ethanol gave the most appropriate quality data for bacterial species identification by routine MALDI-TOF MS.

**FIGURE 3:**
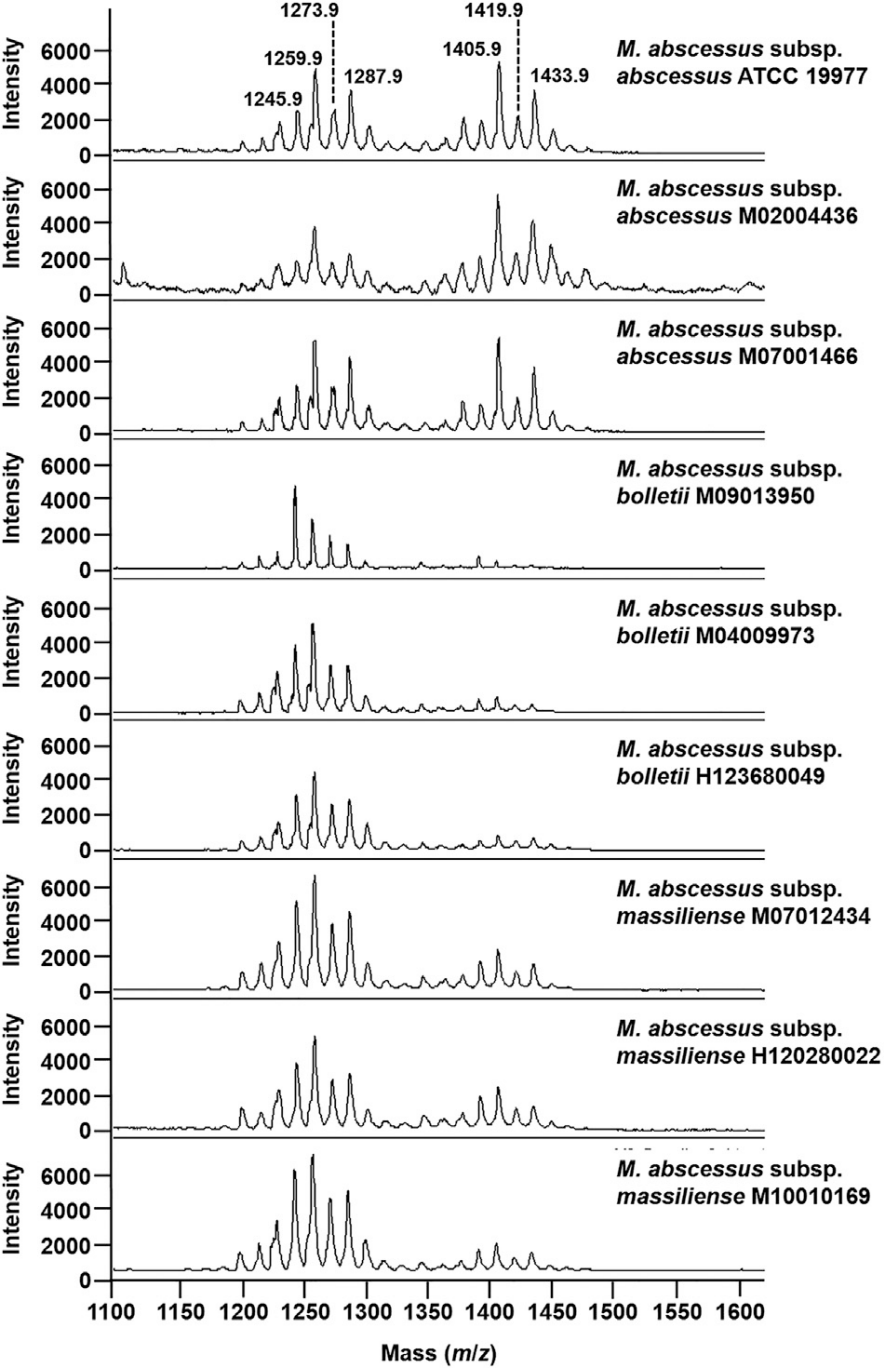
Mass spectra of mycobacteria belonging to the *M. abscessus* complex. Mass spectra were acquired in the positive ion mode with a bacterial suspension set at McFarland 20 and using the super-DHB as a matrix solubilized at 10 mg/ml in 25% ethanol.

To test if that optimized methodological approach has the potential to address the challenge of subspecies typing in the MABS complex, we applied the developed method to *M. abscessus* subsp. *abscessus* ATCC 19977, *M. abscessus* subsp. *abscessus* M02004436, *M. abscessus* subsp. *abscessus* M07001466, *M. abscessus* subsp. *bolletii* M09013950, *M. abscessus* subsp. *bolletii* M04009973, *M. abscessus* subsp. *bolletii* H123680049, *M. abscessus* subsp. *massiliense* M10010169, *M. abscessus* subsp. *massiliense* M07012434, and *M. abscessus* subsp. *massiliense* H120280022. . As seen in Figure 3 for the mass spectra for the *M. abscessus* subsp. *abscessus* strains, the mass spectra for the *M. abscessus* subsp. *bolletii* strains and *M. abscessus* subsp. *massiliense* strains are composed of two sets of peaks starting at *m*/*z* 1201.8 up to *m*/*z* 1299.9 and starting at *m*/*z* 1375.9 up to *m*/*z* 1445.9, distant of 14 mass units with the most intense peaks assigned to potassium cationized–diglycosylated glycopeptidolipids and triglycosylated glycopeptidolipids. However, as opposed to the mass spectrum of *M. abscessus* subsp. *abscessus* strains, where the ratio of diglycosylated glycopeptidolipids/triglycosylated glycopeptidolipids is ∼1:1, in the mass spectra of *M. abscessus* subsp. *bolletii* strains, the ratio of diglycosylated glycopeptidolipids/triglycosylated glycopeptidolipids is ∼1:0.2 and for *M. abscessus* subsp. *massiliense* strains, the ratio of diglycosylated glycopeptidolipids/triglycosylated glycopeptidolipids is ∼1:0.5 (Figure 3). Taken together, those data suggest that the lipid fingerprint has the potential to discriminate between mycobacteria within the MABS complex.

## Conclusion

Here, we provide a new and simple method for the detection of subspecies-specific lipids, applied to the MABS complex. The method is easy to put in place, requires a minimal number of steps, and is performed on a routine MALDI mass spectrometer in the positive ion mode. One of the limitations of the study resides in the use of a very limited amount of strains (*n* = 9). Further studies are now required to test and validate that new approach in the rapid detection of the mycobacteria belonging to the MABS complex, using characterized clinical isolates and other bacterial species and subspecies.

## Data Availability

The original contributions presented in the study are included in the article/Supplementary Material; further inquiries can be directed to the corresponding author.
